# Inter-rater reliability and content validity of the measurement tool for portfolio assessments used in the Introduction to Clinical Medicine course at Ewha Womans University College of Medicine: a methodological study

**DOI:** 10.3352/jeehp.2024.21.39

**Published:** 2024-12-10

**Authors:** Dong-Mi Yoo, Jae Jin Han

**Affiliations:** 1 Department of Medical Education, College of Medicine, The Catholic University of Korea, Seoul, Korea; 2 Department of Medical Education & Thoracic Surgery, Ewha Womans University College of Medicine, Seoul, Korea; Hallym University, Korea

**Keywords:** Clinical medicine, Educational status, Medical education, Reproducibility of results, Republic of Korea

## Abstract

**Purpose:**

This study aimed to examine the reliability and validity of a measurement tool for portfolio assessments in medical education. Specifically, it investigated scoring consistency among raters and assessment criteria appropriateness according to an expert panel.

**Methods:**

A cross-sectional observational study was conducted from September to December 2018 for the Introduction to Clinical Medicine course at the Ewha Womans University College of Medicine. Data were collected for 5 randomly selected portfolios scored by a gold-standard rater and 6 trained raters. An expert panel assessed the validity of 12 assessment items using the content validity index (CVI). Statistical analysis included Pearson correlation coefficients for rater alignment, the intraclass correlation coefficient (ICC) for inter-rater reliability, and the CVI for item-level validity.

**Results:**

Rater 1 had the highest Pearson correlation (0.8916) with the gold-standard rater, while Rater 5 had the lowest (0.4203). The ICC for all raters was 0.3821, improving to 0.4415 after excluding Raters 1 and 5, indicating a 15.6% reliability increase. All assessment items met the CVI threshold of ≥0.75, with some achieving a perfect score (CVI=1.0). However, items like “sources” and “level and degree of performance” showed lower validity (CVI=0.72).

**Conclusion:**

The present measurement tool for portfolio assessments demonstrated moderate reliability and strong validity, supporting its use as a credible tool. For a more reliable portfolio assessment, more faculty training is needed.

## Graphical abstract

[Fig f1-jeehp-21-39]

## Introduction

### Background/rationale

In medical education, portfolio assessment is highly valued for integrating learning with assessment and providing continuous feedback to monitor learners’ improvement [1]. Moreover, portfolios are effective for evaluating complex attributes such as attitudes, personal qualities, and professionalism, which are challenging to assess using traditional methods [2].

However, for portfolio assessment to be fully integrated into the curriculum, issues related to inter-rater reliability and content validity must be addressed. Medical schools often face a general lack of faculty members who can act as raters relative to the number of students. Consequently, portfolio assessments are distributed among multiple raters. If inter-rater reliability is low, the same portfolio might yield inconsistent results depending on the rater, thereby undermining the consistency and fairness of the assessment [3]. Van der Vleuten and Schuwirth [4] highlighted that when inter-rater reliability is not ensured, inconsistencies in scoring among raters can confuse learners, erode trust, compromise the fairness of the assessment, and ultimately limit the usability of the results for educational purposes. Without ensuring content validity, even consistent assessments among raters may fail to provide meaningful insights into learners’ performance and professional growth.

To address these issues, it is essential to minimize judgment errors during the assessment process, apply clear criteria, and ensure reliability and validity through rater training [5,6]. These measures are prerequisites for establishing portfolio assessment as a credible assessment tool. Nevertheless, research analyzing both the inter-rater reliability and content validity of portfolio assessments in medical education remains limited.

### Objectives

This study aimed to examine the inter-rater reliability and content validity of a measurement tool for portfolio assessment used in the Introduction to Clinical Medicine (ICM) course at the Ewha Womans University College of Medicine.

## Methods

### Ethics statement

Ethical approval was obtained from the institutional review board (IRB) of Songeui Medical Campus, the Catholic University of Korea (IRB approval no., MC23EISI0096). The requirement to obtain informed consent was waived by the ethics committee.

### Study design

This study was designed as a methodological study. Data were collected at a single point in time to analyze the content validity (content validity index, CVI) of portfolio assessment items and the inter-rater reliability of portfolio scoring.

### Setting

The study was conducted from September 2018 to December 2018 during the second semester of the first year in the ICM course at Ewha Womans University College of Medicine. This study analyzed a random sample of 5 portfolios from the 64 submitted by students enrolled in the ICM course. The 5 portfolios were selected using a random number generator to ensure unbiased sampling from the total of 64 portfolios. The portfolio assessment involved a gold-standard rater and 6 independent raters, who assigned scores based on predefined assessment criteria (Supplement 1). The 6 faculty raters independently evaluated the portfolios using the standardized assessment criteria. No additional educational interventions or curriculum development occurred during the study period. A panel composed of 7 experts evaluated the content validity of the portfolio assessment items.

### Participants

The raters for portfolio assessment and the expert panel for content validity evaluation were each selected in a way that aligned with the specific purposes of this study.

The raters consisted of 7 individuals, including 1 gold-standard rater who provided benchmark scores as a reference for the remaining 6 raters. The gold-standard rater, selected for their extensive expertise in portfolio assessment and scoring rubric development, was identified following the approach described by Graham et al. [7] for establishing gold-standard ratings in medical education. This rater’s scores served as reference points due to their demonstrated expertise and experience [7]. Excluding the gold-standard rater, the remaining 6 raters were all medical school faculty members with an average of 3 years of experience in portfolio education and assessment. These 6 raters, after receiving prior training on the portfolio assessment criteria, participated in the study.

Separately, the expert panel consisted of 7 faculty members with extensive experience in student education and ICM instruction. This panel was tasked with evaluating the content validity of portfolio assessment items. They rated the appropriateness of the assessment items using a 4-point Likert scale.

### Variables

The outcome variables were values of the Pearson correlation coefficient used to measure the correlation between the scores assigned by the gold-standard rater and the 6 raters, the ICC, and item-level CVI (I-CVI).

### Data sources

Data were collected after the portfolio assessment was completed by requesting individual scores from the raters and expert panel. Personal information such as age, gender, and region was not collected, as it was irrelevant to the study’s objectives.

### Measurement

Scores for 5 portfolio domains were collected from the gold-standard rater and 6 raters. Each score was assigned independently based on the students’ portfolio content, measured on a 100-point scale. Pearson correlation coefficients were calculated to analyze the relationships between the gold-standard rater and individual raters. ICC values were calculated to evaluate overall inter-rater reliability. To evaluate the impact of extreme raters, ICC values were recalculated after excluding their scores. The CVI for each assessment item was calculated based on the expert panel’s responses to the 12 assessment items. Items rated 3 or higher by the panel were considered valid.

### Bias

The gold-standard rater’s scores may have been influenced by subjective judgment. To minimize this bias, the rater was selected based on extensive experience, including over 5 years of portfolio assessment and development of ICM portfolio scoring rubrics.

Differences in rater experience and interpretation of assessment criteria could introduce bias. To address this, all raters received training before the assessment, and ICC was recalculated after excluding extreme raters to evaluate the effect of rater variability.

CVI ratings depend on subjective judgments from experts. To minimize the potential for related problems, only faculty members with substantial expertise in ICM education and portfolio assessment were included in the panel.

### Study size

Sample size estimation was not done due to the limited recruitment of the raters.

### Statistical methods

All statistical analyses mentioned in the measurement section were conducted using IBM SPSS software ver. 26.0 (IBM Corp.).

## Results

### Participants

Table 1 shows the demographic characteristics and areas of expertise of the participants.

### Reliability analysis

#### Rater alignment assessment

Pearson correlation coefficients were calculated between the gold-standard rater and individual raters to assess their alignment and fairness. Rater 1 exhibited the highest average correlation (0.8916), showing strong agreement across all domains, particularly in the “goal domain” (0.9529) and the “processing domain” (0.8618). In contrast, Rater 5 showed the lowest average correlation of 0.4203, with the “reflection domain” (–0.0963) exhibiting the weakest alignment (Table 2).

#### Identification of extreme raters

Based on the average Pearson correlation coefficients, Rater 1 and Rater 5 were identified as extreme raters. Rater 1 had the highest average correlation of 0.8916, suggesting potential mimicry of the gold-standard rater, while Rater 5 had the lowest average correlation of 0.4203, reflecting inconsistency in scoring.

### Inter-rater reliability analysis

To evaluate the overall inter-rater reliability of the scoring system, the ICC was calculated for all raters and after excluding extreme raters. The ICC for all raters was 0.3821, indicating moderate agreement. When Rater 1 and Rater 5 were excluded, the ICC increased to 0.4415, representing a 15.6% improvement in inter-rater reliability (Dataset 1).

### Content validity analysis

The content validity of 12 portfolio assessment items was assessed using the CVI based on expert opinion. All items achieved a CVI of ≥0.75, confirming their validity. Items such as “relationship to the academic goal of ICM,” “self-assessment and reflection,” and “motivation” received the highest score of 1.0, while “level and degree of performance” and “sources” had relatively lower validity, with a CVI of 0.72 (Table 3, Dataset 2).

## Discussion

### Key results

This study evaluated the inter-rater reliability and validity of portfolio assessments in medical education, yielding several key findings. In the analysis of rater alignment, Pearson correlation analysis between the gold-standard rater and individual raters revealed that Rater 1 exhibited the highest correlation (0.8916), while Rater 5 showed the lowest (0.4203). For inter-rater reliability, the ICC for all raters was measured at 0.3821, which increased to 0.4415 after excluding the 2 extreme raters (Rater 1 and Rater 5), demonstrating a 15.6% improvement in system reliability. The content validity analysis of 12 portfolio assessment items revealed that most items (9 out of 12) exceeded the threshold of CVI ≥0.75, with 3 items—“relationship to the academic goal of ICM,” “self-assessment and reflection,” and “motivation”—achieving the maximum score of CVI=1.0. However, lower validity was observed for 2 items: “sources” and “level and degree of performance” (both CVI=0.72).

### Interpretation

This study provides empirical evidence for establishing portfolio assessments as reliable and valid tools in medical education. The improvement in ICC after removing extreme raters underscores the importance of addressing rater variability to improve reliability. Specifically, the increase in ICC after excluding Rater 1 and Rater 5 highlights the critical role of managing the impact of extreme raters in maintaining a consistent assessment system. The high CVI values for items such as “relationship to the academic goal of ICM” and “self-assessment and reflection” demonstrate that these items are strongly aligned with educational objectives. In contrast, items with lower validity, such as “sources” and “level and degree of performance,” indicate the need for clearer and more consistent assessment criteria. These findings suggest that additional training for raters and refinement of the scoring rubrics could help mitigate inconsistencies and improve the validity of the assessment process. The observed negative correlation for Rater 5 in the “reflection domain” (–0.0963) highlights the potential for misinterpretation or excessive subjectivity in certain domains, suggesting that some raters may require additional guidance to align their scoring with established criteria.

### Comparison with previous studies

The findings of this study align with and extend previous research in several key aspects. Our observed ICC improvement after excluding extreme raters (from 0.3821 to 0.4415) is consistent with the findings of Roberts et al. [5], who reported that targeted rater training and careful selection of raters improved reliability coefficients by approximately 20%. The challenges with inter-rater reliability identified in our study echo those found by Driessen et al. [8], who reported ICC values ranging from 0.35 to 0.65 in portfolio assessments across medical schools.

Particularly noteworthy is the alignment of our findings with the comprehensive study of Yoo et al. [9] comprehensive study on portfolio assessment systems in Korean medical schools. Their research, which developed and validated a standardized portfolio assessment system, reported similar challenges in maintaining consistent assessment standards across different raters. Their study emphasized the importance of systematic rater training and clear assessment criteria, which our findings strongly support.

The content validity findings align with Van Tartwijk and Driessen’s [2] comprehensive review of portfolio assessment in medical education, which emphasized the importance of clear assessment criteria. However, our study extends their work by providing specific CVI values for individual assessment items, offering more granular insights into which aspects of portfolio assessment require refinement.

Our observation of extreme rater patterns introduces a new perspective to the literature. While previous studies such as that of van der Vleuten and Schuwirth [4] discussed the importance of rater consistency, our finding that excluding extreme raters can significantly improve reliability offers a practical approach to enhance assessment quality. This builds upon the work of Grant et al. [6] on portfolio assessment reliability but provides a more specific strategy for improving inter-rater reliability.

### Limitations

This study has several limitations. First, the sample size of 5 portfolios and 7 expert panel members was adequate for the study design but may limit the generalizability of the findings. A larger sample size could improve the robustness of the results. Second, the gold-standard scores relied on the subjective judgment of a single rater, which could introduce bias despite the rater’s extensive experience. Third, differences in the raters’ levels of experience were not analyzed, which may have influenced the outcomes.

### Generalizability

Although this study was conducted using data from a single medical school’s ICM course, the findings have broader implications for various educational contexts. Specifically, by focusing on rater consistency and the validity of assessment criteria, the results of this study can inform efforts to improve the reliability and applicability of portfolio assessments in other disciplines requiring robust evaluation frameworks.

### Suggestions for further studies

To improve inter-rater reliability, the development of rater training programs and regular sessions for rater education and feedback are recommended. Items with lower CVI values should undergo a thorough review, incorporating expert feedback to refine assessment criteria and ensure consistency. Expanding future studies to include larger sample sizes and diverse educational environments would provide additional evidence supporting the reliability and validity of portfolio assessments. Future studies should consider incorporating multiple gold-standard raters or a consensus-based scoring approach to minimize the reliance on a single evaluator and further increase the reliability of the gold-standard scores. Additionally, leveraging advanced technology, such as AI-based scoring systems, could provide a supplementary layer of objectivity and consistency in portfolio assessments.

### Conclusion

This study examined the reliability and validity of portfolio assessments, supporting their use as reliable tools in medical education. The improvement in ICC after excluding extreme raters demonstrates a practical approach to enhancing the reliability of the assessment system. Furthermore, more intensive faculty training in portfolio assessment is needed for more consistent assessment. The CVI findings confirmed that the portfolio assessment items aligned well with educational objectives, while also identifying areas for potential refinement. These results highlight the value of portfolio assessments in fostering learner self-reflection and continuing growth, with potential applicability to other professional education settings.

## Figures and Tables

**Figure f1-jeehp-21-39:**
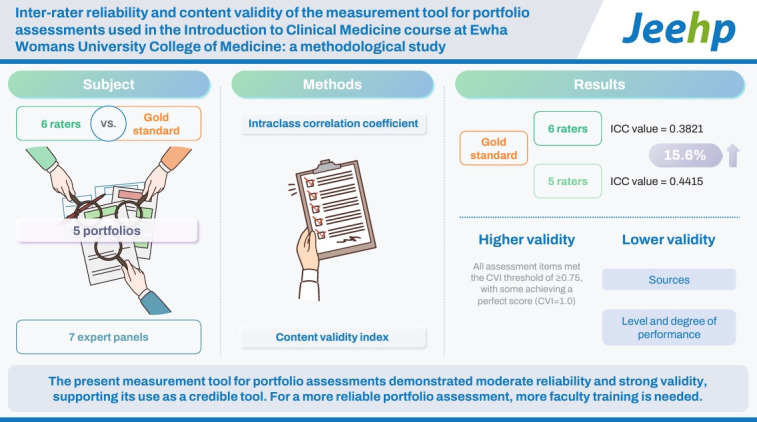


**Table 1.** Demographic characteristics and areas of expertise of the participants

Participant group	Role	No. of participants	Characteristics
Raters	Portfolio raters	6	Faculty members with an average of 3 years of experience in ICM portfolio assessment
Gold-standard rater	Provider of benchmark scores	1	Developed scoring criteria for ICM portfolios and has over 5 years of portfolio assessment experience
Experts	Validators of CVI assessment items	7	Medical school faculty with extensive experience in student education and ICM instruction

ICM, Introduction to Clinical Medicine.

**Table 2.** Domain-wise Pearson correlations between 6 raters and the gold-standard rater

Domain of assessment	Rater 1	Rater 2	Rater 3	Rater 4	Rater 5	Rater 6
Goal	0.9529	0.5616	0.9273	0.6792	0.5093	–0.0707
Overview	0.9595	0.5845	0.7655	0.8014	0.6843	0.8059
Processing	0.8618	0.8605	0.8433	0.0466	–0.0963	0.7530
Reflection	0.7929	0.3590	0.9078	0.3687	0.5838	0.7494

**Table 3.** Content validity index of ICM portfolio assessment criteria

Domain of assessment	Items of assessment	CVI
Goal	(1) Relationship to the academic goal of ICM	**1.0**
	(2) Level of the set goal	**0.86**
	(3) Practicality of the goal	**0.86**
Processing	(1) Relation to the objectives	**0.86**
	(2) Writing of the specific content	**0.86**
	(3) Level and degree of performance	0.72
Reflection	(1) Self-assessment as well as level and degree of self-reflection	**1.0**
	(2) Motivation: effort to improve, will, method, etc.	**1.0**
	(3) Writing of self-study plan and evaluation of the content	**0.86**
Overview	(1) Form	**0.86**
	(2) Description	**0.86**
	(3) Sources	0.72

Bold text indicates CVI ≥0.75. ICM, Introduction to Clinical Medicine; CVI, content validity index.

## Footnotes

**Data availability**
Data files are available from Harvard Dataverse: https://doi.org/10.7910/DVN/ZNTIWJ Dataset 1. Research data for the inter-rater reliability of portfolio assessment.

Dataset 2. Research data for the inter-rater reliability of content validity.

## Supplementary materials

Supplementary files are available from Harvard Dataverse: https://doi.org/10.7910/DVN/ZNTIWJ

Supplement 1. The criteria for “Introduction to Clinical Medicine” portfolio assessment.

Supplement 2. Audio recording of the abstract.
