# Supplementary figures and images for: Inter-rater reliability and content validity of the measurement tool for portfolio assessments used in the Introduction to Clinical Medicine course at Ewha Womans University College of Medicine: a methodological study
Source: J Educ Eval Health Prof. 2024 Dec 10;21:39. doi: 10.3352/jeehp.2024.21.39 (PMC11717432; doi:10.3352/jeehp.2024.21.39)

**Supplement 1.** The criteria for ICM portfolio assessment


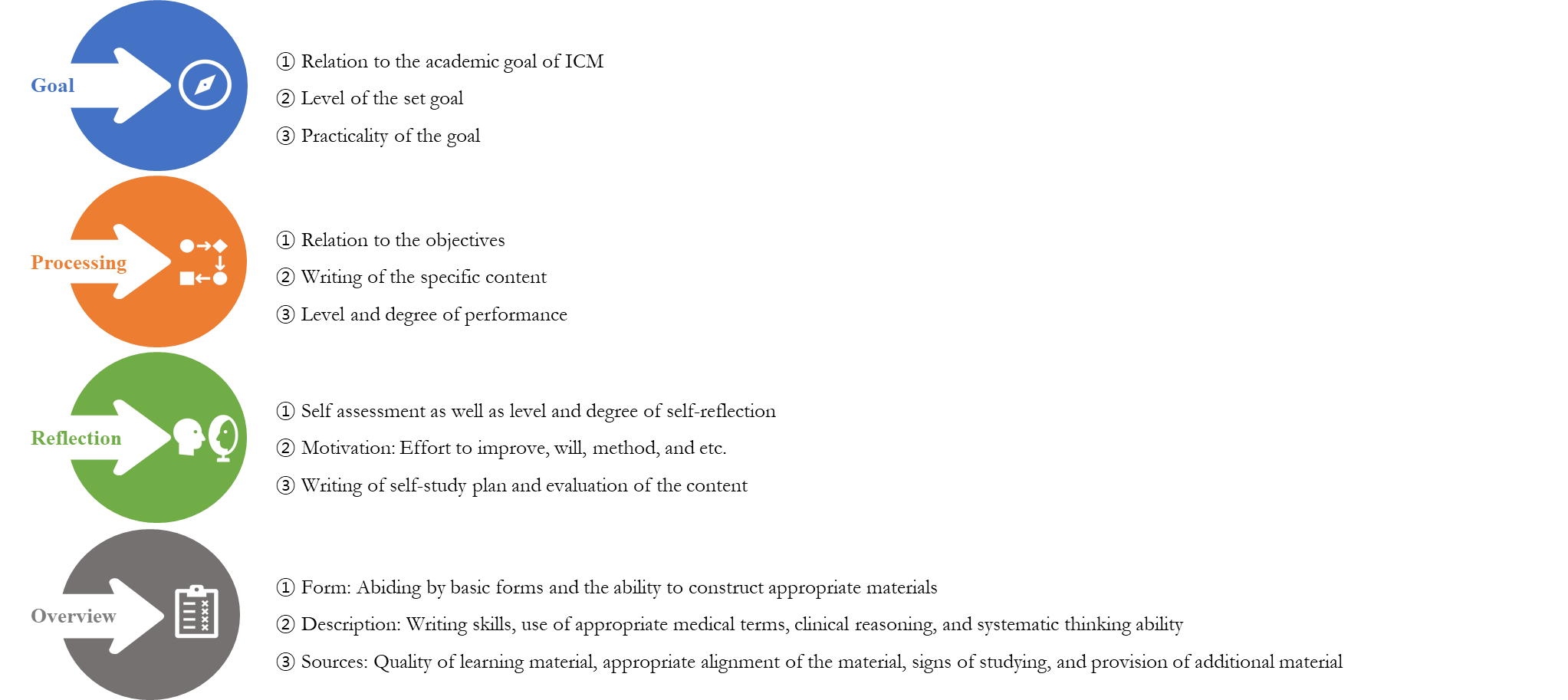

Supplement: Supplementary file 3 — Supplement 1. The criteria for “Introduction to Clinical Medicine” portfolio assessment. [file jeehp-21-39-suppl1.docx]
